# Mixed-methods single-arm repeated measures study evaluating the feasibility of a web-based intervention to support family carers of persons with dementia in long-term care facilities

**DOI:** 10.1186/s40814-018-0356-7

**Published:** 2018-10-31

**Authors:** Wendy Duggleby, Kathya Jovel Ruiz, Jenny Ploeg, Carrie McAiney, Shelley Peacock, Cheryl Nekolaichuk, Jayna Holroyd-Leduc, Sunita Ghosh, Kevin Brazil, Jennifer Swindle, Dorothy Forbes, Sandra Woodhead Lyons, Jasneet Parmar, Sharon Kaasalainen, Laura Cottrell, Jillian Paragg

**Affiliations:** 1 Nursing Research Chair Aging and Quality of Life, Innovations in Seniors Care Research Unit, Faculty of Nursing Level 3, Edmonton Clinic Health Academy, University of Alberta, 11405 87 Avenue, Edmonton, AB T6G 1C9 Canada; 2 Aging, Community and Health Research Unit, School of Nursing, McMaster University, 1280 Main Street West, Hamilton, ON L8S 4K1 Canada; 3 School of Public Health and Health Systems, Schlegel Research Chair in Dementia, Schlegel-UW Research Institute for Aging, University of Waterloo, 200 University Avenue West, Waterloo, ON N2l 3G1 Canada; 4 College of Nursing, University of Saskatchewan, 4340 E-wing Health Sciences104 Clinic Place, Saskatoon, SK S7N 2Z4 Canada; 5 Division of Palliative Care Medicine, University of Alberta, c/o Palliative Institute, 404, Health Services Centre, 1090 Youville Drive West, Edmonton, AB T6L 0A3 Canada; 6 Section of Geriatric Medicine, Cumming School of Medicine, Brenda Strafford Foundation Chair of Geriatric Medicine, University of Calgary, 1403 29th Street NW, Calgary, AB T2N 2T9 Canada; 7 Department of Medical Oncology/Department of Mathematical and Statistical Sciences, University of Alberta, 11560 University Avenue, Edmonton, AB T6G 1Z2 Canada; 8 School of Nursing and Midwifery, Queen’s University Belfast, 97 Lisburn Road, Belfast, BT9 7BL UK; 9 Faculty of Nursing Level 3, Edmonton Clinic Health Academy, University of Alberta, 11405 87 Avenue, Edmonton, AB T6G 1C9 Canada; 10 Arthur Labatt Family School of Nursing, Western University, London, ON N6A 5B9 Canada; 11 Institute for Continuing Care Education and Research (ICCER), 4-023 Edmonton Clinic Health Academy, University of Alberta, 11405 - 87 Avenue NW, Edmonton, AB T6G 1C9 Canada; 12 Department of Family Medicine University of Alberta, CH Network of Excellence in Seniors’ Health and Wellness, Home Living and Transitions, AHS EZ Continuing Care, c/o Grey Nuns Community Hospital, 416 St. Marguerite Health Services Centre, 1090 Youville Drive West, Edmonton, AB T6L 0A3 Canada; 13 School of Nursing, McMaster University, 1280 Main Street West, Hamilton, ON L8S 4K1 Canada

**Keywords:** Web-based intervention, Family carers, Dementia, Long-term care, Hope, Grief, Self-efficacy, Quality of life

## Abstract

**Background:**

Following institutionalization of a relative with Alzheimer disease and related dementias (ADRD), family carers continue to provide care. They must learn to negotiate with staff and navigate the system all of which can affect their mental health. A web-based intervention, My Tools 4 Care-In Care (MT4C-In Care) was developed by the research team to aid carers through the transitions experienced when their relative/friend with ADRD resides in a long-term care (LTC) facility. The purpose of this study was to evaluate MT4C-In Care for feasibility, acceptability, ease of use, and satisfaction, along with its potential to help decrease carer’s feelings of grief and improve their hope, general self-efficacy, and health-related quality of life.

**Methods:**

The study was a mixed-methods single-arm repeated measures feasibility study. Participants accessed MT4C-In Care over a 2-month period. Data were collected at baseline and 1 and 2 months. Using a checklist, participants evaluated MT4C-In Care for ease of use, feasibility, acceptability, and satisfaction. Measures were also used to assess the effectiveness of the MT4C-In Care in improving hope (Herth Hope Index), general self-efficacy (GSES), loss and grief (NDRGEI), and health-related quality of life (SF12v2) of participants. Qualitative data were collected at 2 months and informed quantitative findings.

**Results:**

The majority of the 37 participants were female (65%; 24/37), married (73%; 27/37), and had a mean age of 63.24 years (SD = 11.68). Participants reported that MT4C-In Care was easy to use, feasible, and acceptable. Repeated measures ANOVA identified a statistically significant increase over time in participants hope scores (*p* = 0.03) and a significant decrease in grief (< 0.001). Although significant differences in mental health were not detected, hope (*r* = 0.43, *p* = 0.03) and grief (*r* = − 0.66, *p* < 0.001) were significantly related to mental health quality of life.

**Conclusion:**

MT4C-In Care is feasible, acceptable, and easy to use and shows promise to help carers of family members with ADRD residing in LTC increase their hope and decrease their grief. This study provides the foundation for a future pragmatic trial to determine the efficacy of MT4C-In Care.

**Trial registration:**

ClinicalTrials.gov NCT03571165. June 30, 2018 (retrospectively registered).

## Background

The need to support family carers who are the cornerstone of systems of care and support for persons with Alzheimer disease and related dementias (ADRD) is recognized worldwide [1]. As a result of caregiving, family carers of persons with ADRD experience negative physical and psychological well-being [2]. Families/friends (family carers) continue to provide significant care even after their relative is admitted to long-term care (LTC) [3, 4], and evidence indicates that their mental health might actually worsen after the institutionalization of their family member [5].

In 2011, 4.5% of the Canadian population over the age of 65 years was living in LTC and 60% of these individuals had ADRD [6]. Those admitted to these facilities are frail and vulnerable because of their complex health needs and often require significant healthcare resources [7]. Carers of persons with ADRD residing in LTC experience multiple, complex transitions as a result of changes in roles/relationships, physical and mental health, and hope [8]. Transitions are processes by which individuals deal with significant change by incorporating them into their life [9]. For example, through a process of accepting changes in roles in responsibilities, accessing support and resources and redefining what they considered to be a normal role, caregivers are able to identify positive relationships [9]. Interventions that help carers during transitions are thus essential for their quality of life.

Due to the costs associated with face-to-face interventions, a shift towards technology-driven interventions to support family carers has been occurring [10–12]. Three systematic reviews have been published on Internet-based interventions for family carers of persons with ADRD [10–12]. The most common interventions are websites with information and support on various aspects of caregiving, websites combined with telephone or email support, and websites that involve exchange with other carers online. Boots et al. [11] in their review of 12 studies and Hopwood et al. [12] in their review of 40 studies concluded that multi-component online interventions that were flexible and tailored to the individual resulted in increasing carer well-being. These conclusions are similar to those of reviews of online interventions for family carers of older persons with a variety of diagnoses [13, 14]. While initial evaluations of these interventions have been positive [10–12], whether the participants involved in these studies were caring for a person in the community or residing in LTC was unclear. Moreover, none of the reviewed interventions were developed using transition theory [9] and, as such, might not address the significant changes and resultant processes (transitions) that these carers face.

The authors have worked with the Alzheimer Societies of Alberta/Northwest Territories as well as Brant, Haldimand Norfolk, and Hamilton Halton in Ontario for approximately 5 years to develop interventions to support family carers of persons with ADRD. Based on an adaptation of transition theory [15], our research team has previously developed a flexible multi-component intervention that combines information and interactive activities tailored to family carers of older persons with ADRD living in the community (My Tools 4 Care). My Tools 4 Care (https://www.mytools4care.ca) was recently evaluated using a pragmatic randomized control trial approach [16] and found to have a significant positive impact on the hope of participants in the treatment group compared to an educational control group and from baseline [17]. Hope has also been found to have a significant positive relationship with mental health in this population [8].

The experiences of carers of persons with ADRD in the community and those in LTC appear to share some similarities. For example, family carers of persons with ADRD residing in LTC continue to experience loss [18] and significant changes in hope [3]. However, many differences are also apparent. For example, negative interactions between family carers and LTC staff and poor perceptions of care have a negative impact on family carers [5, 19], potentially resulting in an increased need for healthcare services. Family carers are also concerned about end of life decision-making [16].

My Tools 4 Care was adapted for use by carers of persons residing in LTC facilities through the use of focus groups. Focus group participants (carers of persons with ADRD residing in LTC facilities) were asked to review My Tools 4 Care and make recommended changes. Data from the focus groups highlighted the need for carers to advocate and communicate with LTC staff for the care of their family member and the significant perceptions of grief and feelings of guilt experienced by this population [20]. The stakeholder advisory group and the research team then reviewed these data and worked with ATMIST (our web development partners) to develop My Tools 4 Care-In Care (MT4C-In Care) (https://www.mytools4careincare.ca). Carers were also involved in a pre-pilot of MT4C-In Care before the current study began.

My Tools 4 Care-In Care is a self-administered web-based interactive site consisting of four main sections: (a) about me (an interactive piece were carers can write information about themselves or upload pictures etc.), (b) common changes to expect, (c) frequently asked questions, and (d) resources. Table 1 outlines the key elements of MT4C-In Care.

**Table 1:** Key elements of MT4C-In Care

Section	Content
Introduction	The home page is available publicly on the web. It contains: ▪ Introduction to the toolkit ▪ Login box (email/password) ▪ Registration form to create new account (also asks for demographic info) ▪ Password retrieval process ▪ Link to privacy policy, terms of use, etc. ▪ Tutorials and help files
Section 1: About me	Contains guided evidence-based activities to help carers think about and understand transitions. Activities include understanding their inner strengths, what gives them hope, dealing with guilt, advocating with staff, goals of care, and end of life decision-making. For each activity, users can add formatted text, photos, and attachments.
Section 2: Common changes to expect	Contains information about the types of transitions to expect in all areas of their lives, along with quotes from other carers about their experiences (quotes obtained in a previous research study). This section is read-only.
Section 3: Frequently asked questions	Contains questions suggested by carers who participated in a past research study, and answers provided by experts and practitioners in the field. This section is read-only.
Section 4: Resources	Contains: ▪ Contact information for provincial and national organizations ▪ Space to add additional contacts ▪ Information on where to obtain other relevant books, brochures, and resources ▪ Links to websites containing relevant, evidence-based information, plus a short paragraph explaining the purpose of each website ▪ PDFs of key brochures and a link to streaming video of “Living with Hope”
Additional features	▪ Intuitive and easy to use ▪ Ability to change font size for the whole site ▪ Ability to print any page or send information to others by email ▪ Mobile site also available to users. Mobile site is cross-platform (will work on any smart phone).

### Purpose and aims

The purpose of this study was to determine the feasibility of MT4C-In Care and its potential to benefit carers of persons with ADRD residing in LTC before designing a pragmatic randomized control trial to evaluate its efficacy. Specific objectives were to (a) evaluate MT4C-In Care for feasibility, acceptability, ease of use, and satisfaction and (b) obtain preliminary data on the effectiveness of MT4C-In Care with respect to improving hope, general self-efficacy, and health-related quality of life (HRQoL) and decreasing loss and grief in carers of persons with ADRD residing in LTC.

## Methods

### Design

The study used a concurrent mixed-methods [21], single-arm repeated measures feasibility design and was conducted from June 2016 to May 2017. Thirty-seven carers of older persons with ADRD living in LTC received access to MT4C-In Care over a 2-month period. Participants completed measures of hope [Herth Hope Index (HHI)], general self-efficacy [General Self Efficacy Scale (GSES)], loss and grief [Non-Death Revised Grief Experience Inventory (NDRGEI)], and HRQoL (SF12v2) at baseline, 1 month, and 2 months. At 1 and 2 months, carers also completed a MT4C-In Care checklist intended to evaluate MT4C-In Care for ease of use, feasibility, acceptability, and satisfaction. Qualitative open-ended interviews were conducted at 2 months and informed the interpretation of the quantitative data at the results stage of the study. Ethical approval was obtained from the University of Alberta Health Research Ethics Board (Pro00065220) and operational approval from Covenant Health Research Center.

### Recruitment

Participants were recruited over a 2-month time period (January and February 2017) and involved several strategies. Participants from another study who consented to be contacted for future relevant studies and who had a family member with ADRD residing in LTC were contacted. Other forms of recruitment included newspaper advertisement in two cities in Alberta, the Alzheimer’s Society of Alberta & North-west Territories e-newsletter, and dissemination of study pamphlets to family carers attending the organization’s events. The pamphlets and newspaper advertisements encouraged interested carers to contact a 1–800 number or email if they were interested in participating. If participants met the eligibility criteria, then trained research assistants (RAs) obtained telephone consent to participate in the study.

### Participants and setting

A sample of 40 participants was the target as this is considered sufficient for a feasibility study [22]. Inclusion criteria for study participants were as follows: (a) a family/friend of a person 65 years or older with ADRD residing LTC, (b) carers needed to be 18 years or older, (c) English speaking, and (d) needed to have internet access and a valid email account. All participants lived in Alberta, Canada.

### Data collection procedures

RAs contacted potential participants to explain the study, screen carers for eligibility, and obtain telephone informed consent to participate in the study. All data collection took place over the telephone, with conversations being audio-recorded and ranging in length from 30 to 90 min. RAs directly entered quantitative and MT4C-In Care checklist data into RedCap, a secure online data collection system supported by the University of Alberta. Qualitative interview audio files were stored in a secure shared drive and transcribed verbatim, by an experienced transcriptionist. All files were anonymized by assigning a number to participants and removing any identifying information from the transcripts.

**Fig. 1:**
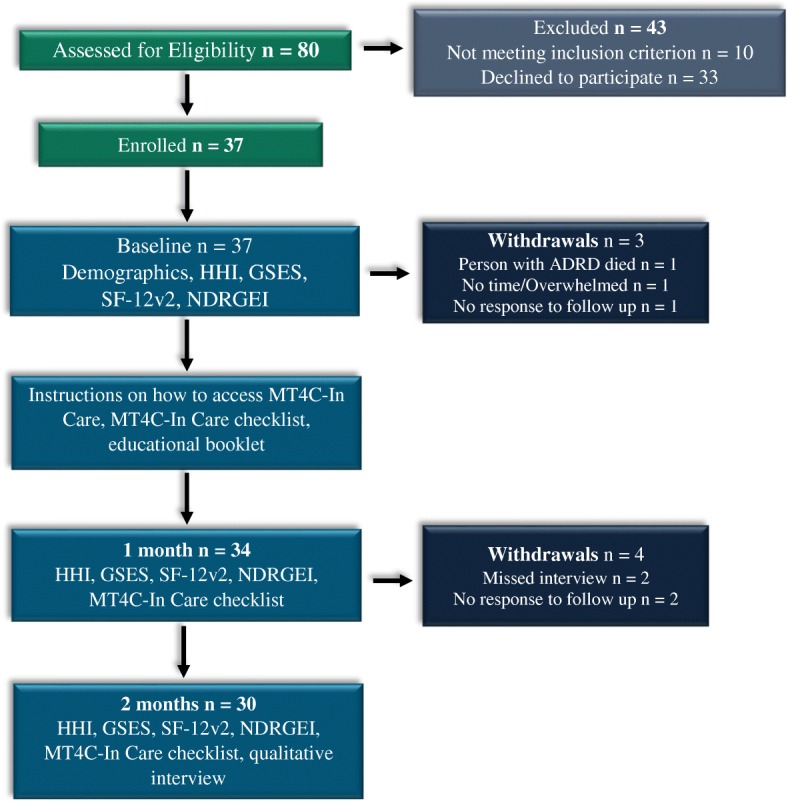
Flow of participants through the study

After consent was obtained, RAs collected demographic data and baseline measures (HHI, GSES, NDRGEI, and SF-12v2) from all participants. All participants then received access to MT4C-In Care and were instructed to use the site at their convenience for 2 months. At subsequent interviews (1 and 2 months), participants completed the same measures along with the MT4C-In Care checklist, which recorded information about participant usage of MT4C-In Care as well as its feasibility, acceptability, ease of use, and satisfaction. At 2 months, all participants were interviewed using semi-structured questions to gather feedback on MT4C-In Care. Figure 1 outlines the data collection procedures for the study.

### My Tools 4 Care-In Care

After the first interview, participants received access to MT4C-In Care for a 2-month period. Research assistants e-mailed the participants’ sign-in information for the MT4C-In Care website, i.e., the website address and a unique username and password; participants were encouraged to change the password at the first log-in. All data entered into MT4C-In Care by participants were kept confidential, in that no one else was able to access their account, including the study team. As MT4C-In Care is a self-administered and flexible intervention tailored to individual use, participants were instructed to access whatever sections they wished and all directions needed for each section are part of the intervention.

### Measures

#### Demographic form

A demographic form was completed by all participants at baseline. Information collected included age, gender, marital status, employment status, ethnicity, income, religion, medical condition, and level of education. Information on the care recipients’ age, gender, and length of time in LTC was also collected.

#### Herth Hope Index

Hope was measured using the HHI, which features a 12-item Likert-type scale [23] with items scored from 1 “strongly disagree” to 4 “strongly agree.” Total scores range from 12 to 48 with higher scores indicating greater hope. The items in the scale can be further divided into three factors: (a) temporality and future, (b) positive readiness and expectancy, and (c) interconnectedness. The HHI has been used in a variety of populations and has a test-retest reliability of 0.91 (*p* < 0.05) and criterion-related validity *r* of 0.81 to 0.92 (*p* < 0.05) [23].

#### General Self-Efficacy Scale

The GSES is a 10-item, 4-point scale with a Cronbach’s alpha coefficient of reliability ranging from 0.76 to 0.90 (*p* < 0.05) [24]. The scale assesses a person’s perceived self-efficacy or their belief that they can complete novel or difficult tasks or cope with diversity. Total scores range from 10 to 40 with higher scores indicating a greater level of self-efficacy.

#### Non-Death Revised Grief Experience Inventory

The NDRGEI is a scale measuring the grief experiences of persons anticipating a loss through four domains: (a) existential concerns, (b) depression, (c) tension and guilt, and (d) physical distress [25]. The NDRGEI is a reliable tool (alpha = 0.93) and consists of 22 items scored on a 6-point Likert scale from slightly disagree to strongly agree, where higher scores are indicative of lower grief responses [26].

#### The 12-item Short-Form Health Survey

The SF-12v2 is the short version of the SF-36 and is a widely used measure of health-related quality of life [27]. The SF-12v2 measures two components of HRQoL: (a) physical health and (b) mental health. Scores range from 0 to 100 with a mean score for the US population of 50 (SD = 10). Higher scores indicate better HRQoL. The SF-12v2 physical component summary (PCS) and mental component summary (MCS) scores strongly correlate with the SF-36, at *r* = 0.95 and 0.97, respectively, and have a good estimated test-retest reliability (PCS: *r* = 0.89; MCS: *r* = 0.76) [28].

#### MT4C-In Care Checklist

This checklist consisted of two parts: (a) use of MT4C-In Care and (b) evaluation of MT4C-In Care. Participants were asked to track their use of MT4C-In Care (i.e., the content accessed and approximate time spent in minutes, at 1 and 2 months). The checklist consists of six Likert-type questions asking participants to rate their agreement with statements from “fully disagree” to “fully agree” regarding the ease of use, feasibility, and acceptability of MT4C-In Care. It also includes a Likert scale to rate overall satisfaction with MT4C-In Care and two qualitative questions asking participants for feedback (“What worked well in MT4C-In Care?” and “What would you do to improve MT4C-In Care?”). The checklist has been used by the authors in previous research [29].

#### Qualitative interviews

The qualitative interview guide, developed by the research team and used in previous research [30], consists of seven open-ended questions intended to evaluate MT4C-In Care. Questions included “What were you thinking about when you worked on MT4C-In Care?”, “Did it help you deal with your significant changes? Why or why not?”, “Did anything influence your ability to work on MT4C-In Care?”, “Who do you think would benefit most from MT4C-In Care?”, “What did you like best?”, “What did you like least?”, and “Anything else you would like to add?”

### Data analysis

Data were analyzed to meet the study objectives of evaluating MT4C-In Care for feasibility, acceptability, ease of use, and satisfaction and to obtain preliminary data on the effectiveness of MT4C-In Care with respect to improving hope, general self-efficacy, and HRQoL and decreasing loss and grief in carers of persons with ADRD residing in LTC.

Quantitative data were checked for accuracy and analyzed using SPSSv24. Descriptive statistics were used to report participants’ demographic characteristics and responses from the MT4C-In Care checklist (feasibility, acceptability, ease of use, and satisfaction). To determine preliminary effectiveness, a repeated measures ANOVA was completed to assess whether changes were present in participants’ HHI, GSES, NDRGEI, and SF-12v2 scores from baseline to 2 months. Because this was a preliminary analysis with a small sample size, co-variates were not used and post hoc analysis was not conducted.

Qualitative data were analyzed using Sandelowski’s [31] descriptive analysis. Each transcript was read, looking for similarities, differences, and patterns in the data and labeling with codes that were then grouped into themes. NVivo 11 software was used to manage and support analysis of the study data. Trustworthiness of the data was maintained by word for word transcriptions that were checked by reviewing audiotapes and ensuring that codes remained data-driven. Audit trails were kept regarding coding decisions through a coding journal. Qualitative data informed the quantitative data in the results stage.

## Results

Seventy of the 80 carers assessed were eligible for the study, with 37/70 (53%) carers of older adults with ADRD living in LTC agreeing to participate in the feasibility study. Seven participants in total withdrew—three (8%) at 1 month and four (11.8%) at 2 months—for reasons that included death of care recipient, lack of time, and no response to follow up telephone calls (see Fig. 1) (retention rate of 30/37; 81.0%). At 1 month, three participants missed their interview with the RA and were unable to re-schedule within the 7-day window allotted to complete the interview. At 2 months, all remaining participants completed their interviews (*n* = 30).

The mean age of carers was 63.2 years (SD = 11.7), with the youngest being 37 and the oldest 86. The majority of carers (65%; 24/37) were female, married (73%; 27/37), Caucasian (81%; 30/37), and the spouse/life partner (30%; 11/37) or adult child (59%; 22/37) of the person with ADRD. On average, carers had completed 15.5 (SD = 3.5) years of education and just under half of participants were employed (49%; 18/37), the majority (55%; 10/18) of which full-time. Care recipients were on average 84.7 years old (SD = 7.4), and the majority were female (76%; 28/37) and had been living in LTC for 37 months (SD = 22.5). Table 2 outlines the demographic characteristics of the study participants.

**Table 2:** Baseline demographic characteristics, *N* = 37

	Mean	SD	*n*	%
Carer				
Age	63.2	11.7		
Years of education	15.5	3.5		
Number of medical conditions	2.4	1.1		
Gender				
Male			13	35.1
Female			24	64.9
Relationship to care recipient				
Spouse/life partner			11	29.7
Son/daughter			22	59.5
Daughter-in-law			2	5
Sister-in-law			1	3
Niece			1	3
Marital status				
Single			4	10.8
Married			27	73
Divorced/separated			4	10.8
Common law			2	5
Ethnicity				
Caucasian			30	81
Chinese			1	3
Southeast Asian			2	5
Other			3	8
No answer			1	3
Actively practicing a religion				
Yes			10	27
No			26	70
No answer			1	3
Employed				
Yes			18	48.6
No			19	51.4
Estimated annual income				
Less than $10,000			0	0
$ 10,000 to $ 39,999			3	8.1
$ 40,000 to $ 69,999			10	27
Greater than $ 70,000			18	48.6
No answer			6	16.2
Do finances meet needs				
Adequately to completely			30	81.1
Totally inadequate to with some difficulty			7	18.9
Care recipient				
Age	84.7	7.4		
Months in 24-h care	37	22.5		
Gender				
Male			9	24.3
Female			28	75.7

### Use of MT4C-In Care

Table 3 presents information about the median time spent in each section of MT4C-In Care, which increased from 1 month to 2 months. The participants spent most of their time at 1 month (19.5 min out of a total of 36 min) and 2 months (95 min out of a total of 132.5 min) on section one, which is the interactive section of MT4C-In Care. At 2 months, participants spent a median time of 30.0 min working on the “Every Day Hope” activity in section one, which was more time than on the other activities in section one and other sections.

**Table 3:** Median time spent on MT4C-In Care toolkit by data collection point

Section: activity	Median time spent in minutes	
	1 month (*n* = 27)	2 months (*n* = 20)
Section 1: Where I am	3.5	15.0
Section 1: What helps me	3.0	10.0
Section 1: My goals as a care partner	2.0	5.0
Section 1: Everyday hope	3.0	30.0
Section 1: What am I doing for myself today?	1.5	10.0
Section 1: How can I manage the guilt that I feel?	2.0	10.0
Section 1: Working together	2.5	7.5
Section 1: Advocating for care	2.0	7.5
Section 1: Total	19.5	95.0
Section 2: Common changes to expect	5.0	10.0
Section 3: Frequently asked questions	6.5	12.5
Section 4: Resources	5.0	15.0
Total time spent	36.0	132.5

### Ease of use of MT4C-In Care

Table 4 presents data related to the MT4C-In Care Checklist ease of use questions. While 31/34 participants completed the checklist at 1 month and 30/30 at 2 months, some participants chose not to answer all the questions in the checklist, for reasons unknown. Data in Table 4 is based on the number of participants who provided a response to each of the questions in the checklist. By 2 months, 92% (23/25) of participants agreed or strongly agreed that the instructions in MT4C-In Care were clear compared to 89% (24/27) at 1 month. By 2 months, 83% (20/24) of participants felt sure about what to do in each activity of MT4C-In Care, and 92% (23/25) reported that MT4C-In Care was easy to use. Qualitative data support these results. For example, one participant stated that:

**Table 4:** Feasibility, acceptability, ease of use, and satisfaction with MT4C-In Care at 1 month and 2 months of data collection

	1 month				2 months			
		Agree to strongly agree	Neutral	Disagree to strongly disagree		Agree to strongly agree	Neutral	Disagree to strongly disagree
Ease of use	*N*		*n* (%)		*N*		*n* (%)	
1. Directions were clear for each activity I wanted to do	27	24 (89)	3 (11)	0 (0)	25	23 (92)	2 (8)	0 (0)
2. I was sure about what to do with each activity I wanted to do	27	21 (78)	5 (19)	1 (3)	24	20 (83)	4 (17)	0 (0)
3. The online format of MT4C-In Care is easy to use	27	25 (93)	1 (3)	1 (3)	25	23 (92)	1 (4)	1 (4)
Feasibility	*N*		*n* (%)		*N*		*n* (%)	
1. I had enough energy to complete each activity I wanted to do	27	17 (63)	4 (15)	6 (22)	24	15 (63)	5 (21)	3 (17)
2. I had enough time to complete each activity I wanted to do	27	16 (59)	4 (15)	7 (26)	24	11 (46)	7 (29)	6 (25)
3. I was able to complete all the activities I wanted to do	27	18 (67)	5 (19)	4 (15)	25	13 (52)	5 (20)	7 (28)
4. The online format of MT4C-In Care is convenient for me	27	25 (93)	1 (3)	1 (3)	26	22 (85)	1 (3)	3 (12)
Acceptability	*N*		*n* (%)		*N*		*n* (%)	
1. MT4C-In Care increased my ability to deal with significant changes	27	16 (59)	7 (26)	4 (15)	24	18 (75)	3 (12)	3 (12)
2. I would recommend MT4C-In Care to someone else	27	25 (93)	1 (3)	1 (3)	25	23 (92)	1 (4)	1 (4)
	1 month				2 months			
		Greatly satisfied	Satisfied	Dissatisfied		Greatly satisfied	Satisfied	Dissatisfied
Satisfaction	*N*		*n* (%)		*N*		*n* (%)	
1. Please tell us how satisfied you were with the toolkit	26	9(35.1)	16(62)	1(2.7)	26	13(50)	10(38)	3(11.5)

MT4C-In Care was easy to use and it gave me awareness of things I hadn’t previously thought of. I think is very well-designed, especially when you consider the demographic: most of the people using this MT4C-In Care will be in their 60’s, so it’s important that it’s so simple.

Despite this, a small number of carers would have preferred MT4C-In Care to be available in a different format: “I am not a computer lover, though. I would rather have it in a book form.”

### Feasibility of MT4C-In Care

Data from the feasibility questions on the MT4C-In Care checklist are presented in Table 4. Most participants agreed that MT4C-In Care was convenient [93% (25/27) at 1 month; 85% (22/26) at 2 months]. This was supported by the qualitative data. For example, one participant said:

It [MT4C-In Care] was the only thing in my life recently that I wasn’t on someone else’s schedule. Like, I could schedule my time to go on there or go there when I had time. ‘Cause most things—most self—things that are—are set up to help people, you have to do it when it’s convenient for them, not when it’s convenient for you.

By 2 months, over half of the participants (63%; 15/24) felt they had enough energy to complete the MT4C-In Care activities they wanted; however, less than half (46%; 11/24) felt they had enough time to do so. Statements in agreement were found in the qualitative data, for example as noted by one participant:

Sometimes when I come back from visiting my wife, depending on how the day has gone, I—I’m quite drained emotionally. You know, I just want to veg out, eh. And, uh—and, uh, so I didn’t get onto it [MT4C-In Care] then.

### Acceptability of MT4C-In Care

Acceptability data are presented in Table 4. Over the course of the study, the number of participants who agreed or strongly agreed that MT4C-In Care helped them with significant changes increased from 59% (16/27) at 1 month to 75% (18/24) at 2 months. When asked how MT4C-In Care had helped them with significant changes, participants said that “it was like a caring person that you were, uh, confiding in.” As one participant noted:

Uh, yeah, I think it does, and the reason being is it makes you think about things. Uh, there’s a lot of thought; you—you look at kind of things and you say, “Oh, I never thought of that,” or “I never thought of this.” You know. Because really, like I say, you’re so emotionally wrapped up in the—in what’s going on, you don’t really—you don’t think logically about where you are and what you should be doing. So, uh, I kind of—kind of like that. Anything that sort of, ah, stimulates your thought process is good, as far as I’m concerned.

MT4C-In Care also “reinforced some of the things I [participant] was trying to achieve. As explained by one participant who felt that MT4C-In Care helped him, “Oh, just helping me come up with ways to communicate without being, um, I do not know, without being abusive or blame.”

While some participants reported that MT4C-In Care did not help them through significant changes, they suggested that was because they were either not experiencing any significant changes or had been carers for some time. However, the majority of carers would recommend MT4C-In Care to someone else [92% (23/25) at 2 months]. Participants also suggested that MT4C-In Care was helpful to carers at all stages of caregiving. For example, in the words of one participant:

I have been doing this for a long time, and went through a lot of this stuff many years ago. I still learned things from the MT4C-In Care, though. The end of life section was very helpful to me.

### Satisfaction with MT4C-In Care

At 1 month, the majority of the participants reported being satisfied with MT4C-In Care (62%; 16/26) (Table 4). Thirty-five percent (9/26) reported being greatly satisfied and only one participant reporting they were dissatisfied. The number of who were greatly satisfied increased to 50% at 2 months (13/26), with 38% (10/26) satisfied and 3/26 dissatisfied. The qualitative data support the satisfaction ratings, as participants made comments such as “[i]t’s a great resource” and “I find the Toolkit extremely helpful, ‘cause it helps me keep on track for myself, but proactively, like, what can I be doing. I’m more grounded and more able.” Participants also described MT4C-In Care as “…. very useful. I—I think it’s very—will be a useful—a very useful tool”.

### Outcome measures

The means and standard deviations (SD) of outcome measures are reported in Table 5. Hypothesis testing was secondary to the feasibility results. Results from the repeated measures ANOVA show a statistically significant increase in participant HHI scores from baseline to 2 months, specifically in the hope sub-factors of positive readiness and expectancy [*F* (2.0, 52.0) = 121.0, *p* < 0.001] and interconnectedness [*F* (1.6, 42.5) = 5.3, *p* = 0.015] (Table 6). A statistically significant decrease in total loss and grief scores was measured by the NDRGEI [*F* (2, 52) = 295.3, *p* = 0.006] and in all four subscales: existential concerns [*F* (2, 52) = 42.5, *p* = 0.003], depression [*F* (2, 52) = 49.3, *p* = 0.021], tension and guilt [*F* (2, 52) = 24.2, *p* = 0.005], and physical distress [*F* (2, 52) = 68.0, p = 0.003]. Due to the small sample size, conducting a post hoc analysis to determine at which time point significant changes occurred was not feasible. Changes in participants’ self-efficacy and HRQoL did not yield statistically significant results. Total HHI (*r* = 0.43, *p* = 0.03) and NDRGEI (*r* = − 0.66, *p* < 0.001) scores were significantly related to MCS (data not shown).

**Table 5:** Descriptive statistics for all outcome measures at baseline, 1 month, and 2 months (rounded to one decimal place)

Measure	Baseline *N* = 37 mean (SD)	1 month *N* = 31 mean (SD)	2 months *N* = 30 mean (SD)
HHI total	38.4 (4.5)	39.8 (3.9)	39.1 (4.2)
HHI Temporality and future	12.6 (1.9)	13.2 (1.9)	13.0 (1.6)
HHI Positive readiness and expectancy	9.9 (1.1)	13.5 (1.3)	13.3 (1.5)
HHI Interconnectedness	12.5 (1.7)	13.0 (1.6)	12.9 (1.7)
GSES total	32.2 (3.8)	32.7 (3.6)	32.5 (3.9)
SF-12v2 PCS	49.5 (12.3)	49.7 (10.7)	50.4 (9.4)
SF-12v2 MCS	49.0 (9.4)	48.4 (9.9)	48.8 (10.4)
NDRGEI total	55.2 (21.1)	50.9 (21.0)	50.2 (21.9)
NDRGEI—existential concern	12.4 (6.2)	14.1 (7.2)	11.5 (6.7)
NDRGEI—depression	16.6 (7.2)	15.2 (6.4)	14.2 (6.8)
NDRGEI—tension and guilt	9.4 (3.8)	7.9 (4.0)	8.2 (4.7)
NDRGEI—physical distress	16.8 (7.1)	13.7 (6.3)	16.4 (7.2)

**Table 6:** Within-subjects effect for all measures at baseline and 1 and 2 months from repeated measures ANOVA*

Measure	Mean square	df	df error	*F*	CI-interval 95%	*P* value
HHI total	32.4	1.5	39.3	4.2	37.4–40.4	0.031*
HHI—temporality and future	3.0	2	52	2.3	12.3–13.5	0.108
HHI—positive readiness and expectancy	121.0	2	52	157.3	11.8–12.7	< 0.001*
HHI—interconnectedness	5.3	1.6	42.5	5.0	12.1–13.3	0.015*
NDRGEI total	295.3	2	52	5.6	43.9–61.0	0.006*
NDRGEI—existential concerns	42.5	2	52	6.6	10.3–15.6	0.003*
NDRGEI—depression	49.3	2	52	4.2	12.8–17.9	0.021*
NDRGEI—tension and guilt	24.2	2	52	5.8	6.9–10.1	0.005*
NDRGEI—physical distress	68.0	2	52	6.6	13.1–18.3	0.003*
GSES total	4.1	2	52	0.7	31.4–33.9	0.520
SF-12v2 PCS	13.4	2	52	0.6	47.1–54.0	0.559
SF-12v2 MCS	4.9	1.6	42.1	0.1	45.1–51.7	0.856

*Significant level set at *p* ≤ 0.05

## Discussion

Participants reported that MT4C-In Care was feasible, acceptable, and easy to use, with these results supported by the qualitative interview data. The majority were also satisfied to highly satisfied with MT4C-In Care. This might be a result of the process used to develop MT4C-In Care, which involved carers of persons with ADRD residing in LTC facilities throughout. The focus groups were instrumental in adapting a previous web-based intervention for carers and ensured the content was appropriate and helpful. Carers were also involved in a pre-pilot of MT4C-In Care to evaluate if the instructions were clear and easy to use. Including potential users in the development process of web-based interventions is a useful process for designing feasible and acceptable interventions [32]. Furthermore, the evaluations of MT4C-In Care at 2 months (with participants reporting higher scores with ease of use and satisfaction at 2 months compared to 1 month) suggests that participants in future evaluations of MT4C-In Care should be asked to use it for a minimum of 2 months. However future research should re-examine if this is the case.

Participants also reported MT4C-In Care helped them deal with the significant changes they experience. Participants described how it encouraged them to reflect on their experience, reinforce their goals as carers, and communicate better with staff. Aligning these comments with the specific activities the participants engaged in would have been useful to determine what aspects of MT4C-In Care were most helpful. As a tailored intervention, participants were instructed to use whatever sections they wanted for as long as they wanted. We used a self-reporting tool to examine what activities/sections of MT4C-In Care were used the most, but data were missing. Similar findings have been reported for other self-administered web-based interventions [17, 29]. Future research should build measures of time spent by each participant on each activity, into the design of the intervention, so that such data would be captured automatically and not rely on self-report. Using this approach, outcomes could be then aligned with specific participants.

Participants reported spending the most time on the hope activity, and the preliminary findings suggest significant increases in hope from baseline. Despite no significant differences in mental health scores from baseline, similar to our study findings, several studies have found a significant positive relationship of hope and quality of life in carers of person with ADRD; as their hope increased so did their mental health [8, 17]. As well, there were significant decreases in grief, in particular, the participants’ tension and guilt. These findings suggest that MT4C-In Care shows promise in achieving positive outcomes for carers of persons with ADRD residing in LTC. Overall, these results are important as MT4C-In Care begins to address the needs of a specific group of carers, namely those caring for a friend/relative with ADRD residing in LTC. Web-based interventions, like MT4C-In Care, can be an accessible, cost-effective means to support carers.

### Limitations

Several limitations to this study relate to participant demographic characteristics and study design. The majority of participants were married, Caucasian women. Women spousal carers of persons with dementia in other studies have been found to have lower HRQoL than men [33, 34]. Moreover, the sample size was small because this was a feasibility study. Future studies should have a larger sample size and include participants with more diverse demographic characteristics.

A power analysis is needed to determine the appropriate sample size to examine the potential efficacy of MT4C-In Care in increasing MCS. Recruitment targets in future research should also calculate the sample size based on an 81% retention rate. This retention rate is relatively high for an intervention study, i.e., once carers started the study, the majority were retained for the 2-month period.

The study design was a single-arm repeated measure design. Without a control or usual care comparison group, preliminary findings of statistical significance should be viewed with caution. Future research designs should utilize a two-arm repeated measure design with a control or usual care group. The preliminary findings (quantitative and qualitative) show that MT4C-In Care has potential benefits and a pragmatic mixed-methods randomized control efficacy trial should be conducted based on the findings of this feasibility study.

## Conclusion

MT4C-In Care appears to be feasible, acceptable, and easy to use, with the majority of participants reporting they were satisfied with the intervention. Preliminary findings support its promise in helping carers deal with their transitions by increasing hope and decreasing feelings of loss and grief, in particular, tension and guilt. Capturing use of MT4C-In Care and detailing the amount of time spent and which sections were used the most is important and could be potentially achieved through the use of a web-based program within MT4C-In Care. Overall, these results provide the foundation for a future full-scale study, specifically a pragmatic mixed-methods randomized control trial with a usual care group that would also analyze the influence of covariates, potentially for mixed-effects modeling of outcomes over the course of the study.

## Abbreviations

ATMIST: Information technology web-developers
GSES: General Self-efficacy Scale
HHI: Herth Hope Index
HRQoL: Health-related quality of life
MCS: Mental component summary
MT4C: My Tool 4 Care
MT4C-In Care: My Tools 4 Care-In Care
NDRGEI: Non-Death Revised Grief Experience Inventory
PCS: Physical component summary
SF-12v2: 12-item Short-Form Health Survey

## References

[1] Prince, M., et al., World Alzheimers Report 2105 The Global Impact of Dementia. Analysis of the prevalanece, incidence, costs & trends. 2015, Alzheimer’s Disease International London, UK.

[2] Papastavrou E (2014). Factors associated with quality of life among family members of patients with dementia in Cyprus. Int Psychogeriatr.

[3] Duggleby, W., D. Schroder, and N. C., Hope and connection: the experience of family caregivers of persons with dementia living in a long term care facility*.* BMC Geriatr, 2013. 13(112): p. 1–8. 10.1186/1471-2318-13-112 PMC4015868 24138640

[4] Mulin J, Simpson J, Froggatt K (2013). Experiences of spouses with dementia in long term care. Dementia.

[5] Gaugler JE (2012). Identifying at-risk dementia caregivers following institutionalization. The nursing home admission-burden and nursing home admission-depression diagnostic tools. J Appl Gerontol.

[6] Statistics and Canada. Living arrangements of seniors. 2015; Available from: http://www12.statcan.gc.ca/census-recensement/2011/as-sa/98-312-x/98-312-x2011003_4-eng.cfm#bx2.

[7] Canadian, et al., When a nursing home is home: how do Canadian nursing homes mesaure up on quality? 2013, CIHI: Ottawa Ontario.

[8] Duggleby W, et al. A mixed methods study of hope, transitions and quality of life in family caregivers of persons with Alzheimer’s disease. BMC Geriatr. 2011;11. 10.1186/1471-2318-11-88 PMC3268103 22192235

[9] Meleis, A., Transitions as nursing theory in Transitions Theory. Middle-range and situation specific theories in nursing research and practice, A. Meleis, Editor. 2010, Springer Publishing Company New York. p. 11.

[10] Godwin KM (2013). Technology-driven interventions for caregivers of persons with dementia: a systematic review. American Journal of Alzheimers Disease and Other Dementias.

[11] Boots LM (2014). A systematic review of Internet-based supportive interventions for caregivers of patients with dementia. International Journal of Geriatric Psychiatry.

[12] Hopwood J (2018). Internet-based interventions aimed at supporting family caregivers of people with dementia: a systematic review. J Med Internet Res.

[13] Andersson S (2017). Information and communication technology-mediated support for working carers of older family members: an integrated literature review. International Journal of Care and Caring.

[14] Ploeg J (2017). Rapid evidence review of the impact of internet interventions on mental health, general caregiving outcomes, and general health for informal caregivers of adults with chronic conditions living in the community. J Med Internet Res.

[15] Duggleby W (2014). Self-administered intervention for caregivers of persons with Alzheimer’s disease. Clin Nurs Res.

[16] Duggleby W, et al. Study protocol: pragmatic randomized control trial of an internet-based intervention (My Tools 4 Care) for family caregivers. BMC Geriatr. 2017. 10.1186/s12877-017-0581-6 PMC5557259 28806917

[17] Duggleby W (2018). Web-based intervention for family carers of persons with dementia and multiple chronic conditions (My Tools 4 Care): pragmatic randomized control trial. J Med Internet Res.

[18] Gaugler JE (2004). Family involvement in nursing homes: effects on stress and well-being. Aging Ment Health.

[19] Peacock Shelley, Duggleby Wendy, Koop Priscilla (2013). The lived experience of family caregivers who provided end-of-life care to persons with advanced dementia. Palliative and Supportive Care.

[20] Cottrell, L., et al., Using focus groups to explore caregiver transitions and needs after placement of family members living with dementia in 24 hour care homes. Aging and Mental Health., Submitted March 14, 2018. 10.1080/13607863.2018.1531369 30588823

[21] Song M, Sandelowski M, and H.M. ISBN:1412972663, Current practices and emerging trends in conducting mixed methods intervention studies in the health sciences*.*, in Sage Handbook of Mixed Methods in Social and Behavorial Research, A. Tashakkori and C. Teddlie, Editors. 2010, SAGE Thousand Oaks ,CA p 725–748.

[22] Lancaster G, Dodd S, Williamson PR (2004). Design and analysis of pilot studies: recommendations for good practice. Journal of Evaluation of Clinical Practice.

[23] Herth Kaye (1992). Abbreviated instrument to measure hope: development and psychometric evaluation. Journal of Advanced Nursing.

[24] Schwarzer R (2012). The general self-efficacy scale.

[25] Lev E, Munor B, McCorkle RA (1993). A shortened version of an instrument measuring bereavemen. Int J Nurs Stud.

[26] Duggleby W, et al. A mixed methods study of hope, transitions and quality of life in family caregivers of persons with Alzheimer’s disease. BMC Geriatr. 2011;11(88). 10.1186/1471-2318-11-88 PMC3268103 22192235

[27] Markowitz JS (2003). Health-related quality of life for caregivers of patients with Alzheimer’s disease. Alzheimer’s Diease and Associated Disorders.

[28] Ware, J.E., et al., User’s manual for the SF-12v2 Health Survey: with a suplement documenting SF-12 Health Survey. 2002, Lincoln, RI QualityMetric Incorporated.

[29] Duggleby W (2017). Feasibility study of an online intervention to support male spouses of women with breast cancer. Oncol Nurs Forum.

[30] Ploeg J (2018). Using a web-based transitions intervention to help informal caregivers of older adults with dementia and multiple chronic conditions: a qualitative study. J Med Internet Res.

[31] Sandelowski M (2010). What’s in a name? Qualitative description revisited. Res Nurs Health.

[32] Dam, A., et al., Development and feasibilty of Inlife: a pilot study of an online social support intervention for informal caregivers of persons with dementia PLoS One, 2017. 12(9): p. e0183386. 10.1371/journal.pone.0183386 PMC5590823 28886056

[33] Jovel-Ruiz, K., W. Duggleby, and A. Williams, Quality of life: caregivers of persons with dementia and multiple chronic conditions*.* International journal of care and caring. , Resubmitted June 27, 2018.

[34] Gibbons C (2014). The psychological and health consequences of caring for a spouse with dementia: a critical comparison of husbands and wives. Journal of Women & Aging.

